# Targeting condom distribution at high risk places increases condom utilization-evidence from an intervention study in Livingstone, Zambia

**DOI:** 10.1186/1471-2458-12-10

**Published:** 2012-01-05

**Authors:** Ingvild Fossgard Sandøy, Cosmas Zyaambo, Charles Michelo, Knut Fylkesnes

**Affiliations:** 1 Centre for International Health, University of Bergen, Bergen, Norway; 2 Department of Public Health, School of Medicine, University of Zambia, Lusaka, Zambia

## Abstract

**Background:**

The PLACE-method presumes that targeting HIV preventive activities at high risk places is effective in settings with major epidemics. Livingstone, Zambia, has a major HIV epidemic despite many preventive efforts in the city. A baseline survey conducted in 2005 in places where people meet new sexual partners found high partner turnover and unprotected sex to be common among guests. In addition, there were major gaps in on-site condom availability. This study aimed to assess the impact of a condom distribution and peer education intervention targeting places where people meet new sexual partners on condom use and sexual risk taking among people socializing there.

**Methods:**

The 2005 baseline survey assessed the presence of HIV preventive activities and sexual risk taking in places where people meet new sexual partners in Livingstone. One township was selected for a non-randomised intervention study on condom distribution and peer education in high risk venues in 2009. The presence of HIV preventive activities in the venues during the intervention was monitored by an external person. The intervention was evaluated after one year with a follow-up survey in the intervention township and a comparison township. In addition, qualitative interviews and focus group discussions were conducted.

**Results:**

Young people between 17-32 years of age were recruited as peer educators, and 40% were females. Out of 72 persons trained before the intervention, 38 quit, and another 11 had to be recruited. The percentage of venues where condoms were reported to always be available at least doubled in both townships, but was significantly higher in the intervention vs. the control venues in both surveys (84% vs. 33% in the follow-up). There was a reduction in reported sexual risk taking among guests socializing in the venues in both areas, but reporting of recent condom use increased more among people interviewed in the intervention (57% to 84%) than in the control community (55% to 68%).

**Conclusions:**

It is likely that the substantial increase in reported condom use in the intervention venues was partially due to the condom distribution and peer education intervention targeting these places. However, substantial changes were observed also in the comparison community over the five year period, and this indicates that major changes had occurred in overall risk taking among people socializing in venues where people meet new sexual partners in Livingstone.

**Trial registration:**

ClinicalTrials.gov NCT01423357.

## Background

In order to design and implement effective HIV prevention interventions, it is essential to have a broad understanding of the epidemiological context in a community. This includes determining whether the HIV epidemic is generalised or concentrated, major modes of transmission, groups with the highest incidence and sexual risk taking in different subpopulations [1,2]. One tool in the assessment of specific epidemiological contexts is the "Priorities for Local AIDS Control Efforts" (PLACE)-method, an approach to rapidly identify places with a high risk of HIV transmission. The underlying assumptions of the method are that risky behaviours often take place in venues which are publicly available, and that targeting HIV preventive activities at such places is likely to be more effective than targeting interventions just at perceived high risk groups in settings with high HIV prevalence. In countries where HIV is primarily heterosexually transmitted, high risk places are defined as places where people meet new sexual partners [3].

There are sharp geographical differentials in HIV prevalence trends in Zambia. Data from pregnant women indicate overall national declines among urban and rural participants since the mid 1990s. However, in certain antenatal surveillance sites, HIV prevalence has been stable. In Livingstone in Southern province the HIV prevalence was stable among young women around 30% during the period 1994-2002 [4] despite high investments in HIV prevention since the early 1990s. In 2005 a PLACE-survey was conducted in Livingstone in order to assess the need for and presence of HIV preventive interventions targeting high risk places. This survey found that 43% of the places where people met new sexual partners never had condoms available. People socializing in the venues reported multiple partners in the previous month, but less than half reported using condoms consistently with new partners. Reported condom use was higher in places where condoms were always available, and this is in line with findings from other studies [5-9]. Many of the respondents expressed a wish to discuss HIV with health personnel or peer educators, but they were afraid to go to the clinic since people might suspect that they were infected [10].

A number of peer educators had previously been trained in Livingstone, but most of them had dropped out due to lack of incentives [10]. Since the survey indicated a high potential for improvement of condom availability, we intended to test whether distributing condoms to places where people met sexual partners could increase condom use among those socializing in these places. We also aimed to assess the operational feasibility of engaging peer educators in condom distribution and behaviour change discussions.

## Methods

### Description of study sites

Livingstone is situated close to the Zimbabwean border, and had a population size of 142,000 in 2010. Maramba is a high density township and many of its 39,000 inhabitants fetch drinking water from communal taps and access communal toilets. More HIV preventive projects have been organized in this township than in other parts of Livingstone because it has been perceived to be a particularly high risk area for HIV. Dambwa is a medium density township with some smaller high density parts and a total population size of 50,000 in 2010 [11].

### Baseline survey

A PLACE-survey was conducted in Livingstone in 2005 to identify high risk places [10]. During the first phase of the survey the interviewers walked through the streets of Maramba, Dambwa and the city centre and asked taxi drivers, bar workers, shop staff, health personnel, and young people to name places where people met new sexual partners. The second phase consisted of locating all the mentioned venues. In the places that were found and in operation, the owner, a bar worker or a regular guest was interviewed about activities taking place in the venue and the availability of condoms, posters and leaflets relating to HIV prevention. During the third phase of the study, all the venues mentioned by more than 10 informants in the first phase and 1/3 of the remaining sites were selected (with probability of selection proportional to the estimated number of guests during busy hours). Individuals socializing in these venues and who were standing along two imaginary diagonal lines connecting the four corners of the room were approached for an interview about sexual behaviour. One nurse, one counsellor and 1-2 peer educators from health clinics in Maramba and Dambwa and staff of NGOs involved in HIV prevention activities in Livingstone were interviewed about HIV preventive programs which existed in the city.

### Intervention

Youth peer educators who were working at the health clinics in Livingstone or had been involved in other peer education activities were invited to participate in the intervention study. The only selection criteria were willingness to visit venues where people meet sexual partners several times per month for a small compensation and ability to communicate with strangers about HIV, sex and condoms. A two-day training was held in February 2008 for 48 youths. A second training/retraining which included 24 new youths was conducted in August of the same year. The training was based on materials and teaching methods developed by the WHO, the Ministry of Health and different NGOs, but was adopted to the specific context, i.e. focusing on condom demonstration and communication with drunk people. To compensate for drop-outs, 11 young persons were recruited in the course of the intervention and given one-to-one training from the two local supervisors who were adults with extensive experience with coordinating youth peer educator activities. Monthly meetings were held between the peer educators and the local supervisors. The supervisors recorded the reasons given for withdrawing when peer educators dropped out.

Maramba was selected as the intervention community because most of the active peer educators came from this township. The list of venues from 2005 was continually updated during the intervention year as new places were established and others closed down. The owners and staff of the listed venues were requested to allow peer educators to bring condoms to the venues and to talk to guests about HIV-related issues. The youth peer educators were given responsibility to distribute condoms and to check the condom stock twice a week in 1-2 venues each. They were told that it was very important that there were always enough condoms available in the venues. Boxes of condoms were to be put in a suitable place, but keeping in mind that people were likely to prefer picking up condoms in a private spot. No specific guide was given concerning what time of the day the visits should be conducted. During visits to the venues the peer educators were supposed to approach the guests about HIV-related issues and to demonstrate condom use. According to the initial plan they would also put up HIV-related posters and distribute leaflets, but this ended up being done irregularly due to a limited budget to produce such materials. Since some of the peer educators did not live within Maramba, a transport allowance equivalent to 4 USD was given for each of the two weekly visits. The peer educators reported their weekly activities, including number of visits and time spent in the venue, number of condoms, posters and leaflets distributed, and number of persons talked to, to the local supervisors in a form called "Peer educator diary".

The intervention ran from the 1st February 2009 to the 31st March 2010. Condoms were obtained from the Livingstone District Health Management Team (DHMT), Livingstone General Hospital and Planned Parenthood Association Zambia during the first 5 months of the intervention, and from the Medical Stores Limited during the remaining period. Excluding the costs of the condoms, the intervention cost 63,400 USD, of which 18,796 USD covered travel and accommodation expenses of the researchers.

An independent person was engaged as an external monitor to assess the presence of condoms, HIV-related posters and leaflets and to find out from staff whether peer educators had engaged in behaviour change discussions. She visited all the venues that were targeted by the intervention on average three times during the intervention year. The identity of the monitor was kept secret to the peer educators and her schedule was unknown to the peer educators, the local supervisors and the research team. The local supervisors discussed shortcomings that were highlighted in her reports with the peer educators at the following monthly meeting.

### Evaluation

In March 2010, a new survey was conducted in Maramba and Dambwa. Since the city centre is in-between the two townships it functioned as a geographical corridor separating the two communities. In Maramba the list of venues included in the intervention was utilized, whereas in Dambwa, phase one was repeated to make an updated list of venues. In the follow-up survey, all the listed venues were visited in both the second and third phases of the survey. The same questionnaires as in the baseline were used in addition to some new questions on experiences relating to HIV prevention interventions in the venues.

In addition, eight peer educators, the two local supervisors and the external monitor were interviewed in-depth (IDI) using a semi-structured interview guide. Two focus group discussions (FGDs) with peer educators (one with 8 females and one with 9 males) were also conducted based on a semi-structured discussion guide. In this paper we focus on the sections of the interviews/discussions relating to experiences with the intervention itself. The peer educators motivation and reasons for dropping out will be the focus of a separate paper. Brief IDIs were also conducted with nine bar attendants and nine male patrons in selected intervention venues touching upon HIV prevention available in the venue. All the interviews and discussions were transcribed verbatim.

### Analyses

The quantitative data was entered in Epidata. The statistical analyses were conducted primarily using SPSS version 15, but StataIC 10 was also utilized. The data collected in the city centre in the baseline survey was not included in the analysis of this paper. The analyses of data from individuals socializing in the venues were adjusted for the effects of clustering (venues were regarded as clusters) and weighted to compensate for differential probability of being selected in popular versus less popular venues. Although the age of respondents and number of partners were not normally distributed, we used the mean and its SE as the main measure of average since we could not compare medians while including frequency weights. However, the independent samples *t*-test used to compare differences in means between subgroups could not be adjusted for the effect of clustering. Differences in percentages between Maramba and Dambwa and between the baseline and follow-up surveys were assessed with the Pearson chi-square test of independence. The significance level was set at 5%. To test whether observed differences were due to differences in the types of venues included in phase 2 or to differences in types of venues, and in age and gender of respondents included in phase 3, adjusted logistic (for binary outcomes) and ordinal (for ordinal dependent variables with more than two response-categories) regression analyses were conducted with township or year as the main independent variable. The age-adjustment was done with a categorical variable. We comment on the results of the adjusted regression analyses only when the adjustments changed the association between the dependent and the main independent variable from significant to non-significant or the other way around. The analyses were done both stratified by gender and pooled. The Pearson correlation coefficient was used to assess the relationship between the reported frequencies of visits by peer educators versus venue representatives.

The qualitative analysis was guided by the framework approach [12]. This approach included reading through all the interviews and discussions and labelling the sections relating to experiences with the PLACE intervention according to predetermined subthemes: frequency and timing of visits, description of typical visits, experiences with condom distribution, experiences with poster distribution, and interactions with guests. The data was grouped and sorted according to the subthemes in a chart, where each case or FGD formed a separate row. The chart was used to map the range of experiences with the intervention.

### Ethical aspects

Only adults aged 18 years and above were interviewed. Oral consent was required of all participants. The interviews were anonymous, and the informants were assured that the information given would not be linked to the site or to them. The interviews with individuals socializing in the venues were conducted in a private corner of the venue or outside, depending on what the respondents preferred. Both the baseline and the intervention protocols were approved by the Research Ethics Committee of the University of Zambia.

## Results

### Participation

Fifty eight venues in the intervention community were included at some point during the intervention, but since some closed and new places opened, the number of venues included concurrently varied between 45 and 49. One of the venues was dropped from the intervention during the first month of the intervention because the owner decided not to participate due to religious reasons.

The age of the peer educators ranged between 17-32, with 22 being the average. Forty percent of the peer educators were females. Out of the 72 peer educators trained before the intervention, 15 withdrew before it started and 23 dropped out at some point during the intervention. The most common reasons given were employment (seven), attending school (five), moving out of town (nine), and being discouraged by late or low allowances (nine).

In the baseline survey in 2005, 434 persons were asked to name places where people met new sexual partners. Among those mentioned, 55 were located in the intervention community and 71 in the control community. The majority of venues were sherbeens (informal drinking places serving alcohol without a license). The rest were night clubs, hotels and guest houses. During the follow-up survey in 2010, 130 people in the control community were asked to name venues where people met new sexual partners during phase one, and 53 places were mentioned. All of these were visited in phase two. Twelve of the intervention venues were closed permanently or temporarily at the time of the follow-up survey. The majority of the venues in both communities in 2010 were bars (Table 1). In the third phase, 190 and 339 individuals socializing in 25 and 34 selected venues in the intervention and the control community were approached for an interview in the baseline survey, whereas the corresponding numbers were 264 and 273 individuals in 43 and 50 venues, respectively, in the follow-up survey. A few refusals were recorded in the baseline (unweighted 3.8%), whereas no refusals were recorded in the follow-up survey (Table 2).

**Table 1:** Characteristics of and activities in venues where people meet new sexual partners

		Baseline					Follow-up				
		**Intervention**		**Control**			**Intervention**		**Control**		

		**%**	***N***	**%**	***N***	**p-value**	**%**	***N***	**%**	***N***	**p-value**

Place verification	Found	74	*55*	75	*71*	0.388	74	*58*	100	*53*	0.002
	Found, refused interview	2		0			0		0		
	Not found	14		11			0		0		
	Closed temporarily	0		0			7		0		
	Closed permanently	2		0			14		0		
	Not visited	7		14			5		0		

Type of place	Sherbeen	55	*42*	60	*53*	0.439	26	*43*	15	*52*	0.042
	Bar/restaurant	36		36			60		83		
	Night club	5		4			5		2		
	Hotel/guest house	5		0			9		0		

Gender respondent	Male	74	*42*	62	*53*	0.233	37	*43*	57	*53*	0.059
	Female	26		38			63		43		

Position of respondent	Staff	57	*42*	54	*52*	0.749	74	*43*	87	*53*	0.122
	Patron	43		46			26		13		

Refused interview		2	*42*	0	*53*	0.259	0	*43*	0	*53*	-

Beer drinking		95	*41*	100	*53*	0.104	98	*43*	100	*53*	0.264

Spirits drinking		71	*41*	62	*53*	0.305	58	*38*	76	*50*	0.071

Dancing		56	*41*	45	*53*	0.298	93	*42*	94	*48*	0.865

Men meet new female sexual partners here		78	*41*	79	*53*	0.888	100	*43*	92	*52*	0.063

Women meet new male sexual partners here		78	*40*	79	*53*	0.839	98	*43*	90	*52*	0.146

Men meet new male sexual partners here		0	*41*	0	*52*	-	21	*43*	15	*52*	0.483

Women come to sell sex		78	*41*	76	*53*	0.770	93	*43*	73	*52*	0.012

**Table 2:** Socio demographic characteristics of individuals socializing in venues where people meet new sexual partners

	Men									
	**Baseline**					**Follow-up**				

	**Intervention**		**Control**			**Intervention**		**Control**		

	**%**	***N***	**%**	***N***	**p-value**	**%**	***N***	**%**	***N***	**p-value**

Refusals	3.3	*155*	1.3	*261*	0.172	0	*199*	0	*222*	-

Single						30	*198*	45	*221*	0.003
Married/cohabiting						61		50		
Divorced						8		2		
Widowed						1		3		

From township	82	*148*	63	*254*	0.019	88	*199*	91	*220*	0.392
Another township	16		36			8		8		
From out of town	1		2			4		1		

	**Est**	**95% CI**	**Est**	**95% CI**	**p-value**	**Est**	**95% CI**	**Est**	**95% CI**	**p-value**

Median age	29		30			31		30		

Mean age	28.6	27.8-29.3	30.8	29.3-32.3	< 0.001	32.4	30.8-34.1	31.0	29.7-32.2	< 0.001

Median no school years	12		12			11		12		

Mean no school years	11.2	10.9-11.6	11.0	10.2-11.7	0.025	10.4	9.9-11.0	11.6	11.1-12.1	< 0.001

	**Women**									

	**Baseline**					**Follow-up**				

	**Intervention**		**Control**			**Intervention**		**Control**		

	**%**	***N***	**%**	***N***	**p-value**	**%**	***N***	**%**	***N***	**p-value**

Refusals	2.9	*33*	3.1	*77*	0.953	0	*65*	0	*51*	-

Single						41	*65*	56	*51*	0.264
Married/cohabiting						16		24		
Divorced						22		17		
Widowed						20		4		

From township	83	*31*	76	*70*	0.537	96	*65*	94	*51*	0.796
Another township	17		24			3		5		
From out of town	0		0			1		1		

	**Est**.	**95% CI**	**Est**.	**95% CI**	**p-value**	**Est**.	**95% CI**	**Est**.	**95% CI**	**p-value**

Median age	24		24			30		28		

Mean age	24.0	22.5-25.5	25.6	23.7-27.6	0.002	29.8	27.1-32.5	27.5	25.2-29.8	< 0.001

Median no school years	10		9			9		9		

Mean no school years	10.4	9.7-11.0	9.1	8.4-9.8	< 0.001	9.1	8.5-9.6	10.1	8.7-11.6	< 0.001

### The intervention process

On average, each venue was visited 1.6 times per week by peer educators, and they reported speaking to a median of two persons about health and HIV-related issues during a visit. Most visits were conducted on weekdays between 0900 and 1100 hours. Only 9% of the visits were conducted after 1700 and less than 5% on Saturdays or Sundays. However, according to the venue representatives, the busiest hours in the venues were typically between 1700 and 2200 hours, and Friday, Saturday and Sunday were the busiest days. A typical reason given by the peer educators in the in-depth interviews for the visiting hours was that they had other engagements (e.g. work) later in the day, and for those who lived outside the intervention community evening visits were more difficult due to security concerns and less transport being available. In the interviews most of the peer educators reported placing the condom boxes on the counter. Some also mentioned that condoms were placed in the toilets. Bar attendants in two of the guest houses reported that they provided them in the rooms or gave them directly to women who were selling sex. Both the peer educators and the local supervisors reported that some bar attendants did not display the condoms, possibly because they were selling socially marketed condoms.

The peer educators reported that many of the guests were eager to learn about condoms and that the guests usually took the initiative to the discussions. Although some people in the beginning accused the peer educators of promoting promiscuous behaviour, there were less critical voices as time went on and the peer educators explained the purpose of the intervention.

"This time they've accepted the study. So, we're welcome to each and everyone. Any person you find in the venue say, "Uh, madam after you - when you finish talking to the bar-man, you come here. Me, it's me. You're going to start with me. There's something I want to find out more from you". So it's like they - they accepted us." (Female peer educator aged 32, IDI 2)

The peer educators reported meeting a lot of people who expressed appreciation for the free condom distribution. The local supervisor also perceived that the bar owners were happy with the intervention.

"Some of the bar owners - those ones whereby now we've felt that relationship when they meet you- they would talk about it to say it is really helping them. Because you'd find that - that time maybe when the customer needs to run out from the bar to go and look for a condom, he wouldn't go because it's nearer and he'll like boost their business."(Local supervisor, female nurse aged 39.)

However, seven out of the eight peer educators who were interviewed stated that it was difficult to talk to older people about condom use.

"In some cultures it is not allowed for a youth like me to be talking to someone older about sex and condoms. Not just - it's like you are disrespecting them. (...)" (Female peer educator aged 23, IDI 3)

In some bars there were complaints from the bar attendants that the peer educators only left the condoms and did not make an effort to start behaviour change discussions. The external monitor and the peer educators were of the impression that this occurred more often with peer educators who joined the project late and had not received the initial training. At the same time the high turnover of staff in the venues was a challenge mentioned by the peer educators since it meant that new bar attendants had to be sensitized to understand the idea behind the intervention and why condoms should be clearly displayed.

### Monitoring

The external monitor carried out the first monitoring round in May 2009 and found that 26% (10 out of 39) of the visited venues did not have condoms available. In the next two monitoring rounds this was down to 8% (3 out of 36 visited venues in June and 2 out of 24 in July), and then increased to 11% (2 out of 18 visited venues) in August and October and 12% (2 out of 16 visited venues) in the December round. Posters were observed in 23% of the venues in May, 53% in June, 62% in July, 28% in August, 6% in October and 12% in December.

### Impact

The percentage of venue representatives who reported that HIV prevention activities had ever taken place increased sharply in the intervention community between 2005 and 2010 (22% to 91%, p < 0.001), but there was only a slight increase in the control community (21% to 30%, p = 0.280). There was a small, but significant, increase in the proportion of venues in the control community where peer education had taken place (0 to 8%; p = 0.036), and there was a clear increase in condom distribution and peer education in the intervention community. However, the frequency of visits reported by peer educators themselves did only weakly correlate with the frequency reported by venue representatives in the intervention community (Pearson correlation 0.240; p = 0.131). In both the intervention and control communities there was an increase in the proportion of venues where condoms were reported to be always available, but the increase in the control community was not significant when adjusted for the type of sites included. In the follow-up survey, HIV-related leaflets and posters and condom distribution were all more likely to be observed by the interviewers in the intervention than the control community. All the venues in the intervention community provided condoms free of charge to the guests in 2010 whereas only a quarter in the control community did the same. The free condoms were reported to be provided by the district or NGOs in both townships (Table 3).

**Table 3:** HIV prevention activities in venues where people meet new sexual partners

		Baseline					Follow-up				
		**Intervention**		**Control**			**Intervention**		**Control**		

		%	*N*	%	*N*	**p-value**	%	*N*	%	*N*	**p-value**

Ever HIV prevention activities in venue		22	*41*	21	*42*	0.888	91	*43*	30	*50*	< 0.001

Ever HIV-related lectures/seminars in venue		2	*41*	2	*53*	0.854	14	*43*	6	*50*	0.196

Ever HIV-related pamphlets/leaflets in venue		2	*41*	0	*53*	0.253	19	*43*	2	*50*	0.007

Ever HIV-related posters in venue		15	*41*	15	*53*	0.950	26	*43*	4	*50*	0.003

Ever condom distribution in venue		10	*41*	21	*53*	0.149	84	*43*	20	*50*	< 0.001

Ever peer education in venue		0	*41*	0	*53*	-	46	*43*	8	*50*	< 0.001

How often condoms available	Always	32	*41*	17	*52*	0.036	84	*43*	33	*51*	< 0.001
	Sometimes	32		19			16		35		
	Never	37		64			0		31		

Where do you obtain condoms that available to people who come to this venue?	Buy them						0	*43*	58	*43*	< 0.001
	Obtain from NGO						95		14		
	Obtain from district						2		26		
	Other						2		2		

Condom free of charge to guests							100	*43*	24	*42*	< 0.001

Condoms at time of visit		49	*41*	24	*53*	0.015	86	*43*	56	*52*	0.001

If yes, Can I see one?		100	*19*	100	*13*	-	97	*37*	100	*28*	0.381

Posters observed by interviewers		29	*41*	21	*53*	0.341	15	*41*	2	*53*	0.020

Leaflets observed by interviewers		0	*41*	0	*53*	-	2	*41*	0	*53*	0.253

Willingness among staff to distribute free condoms							100	*31*	98	*43*	0.393

Willingness among staff to sell condoms?		88	*24*	70	*27*	0.138					

**Figure 1:**
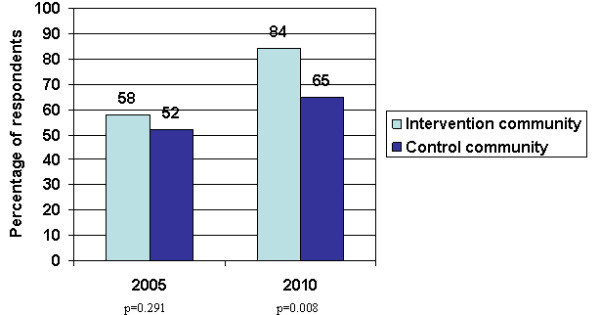
**Proportion of respondents reporting having used a condom with the previous new partner**.

Just over half the respondents in the baseline survey reported having used a condom with the previous partner from the venue where they were interviewed, and this increased significantly to 82% in the intervention community (p = 0.001) and non-significantly to 68% in the control community (p = 0.118). In 2010, 80% of the respondents in the intervention community who reported having used a condom with the previous partner from that venue had obtained it in the same venue, whereas this was the case for less than a quarter in the control community (p < 0.001). This condom was more likely to have been free in the intervention than in the control venues (reported by 72% vs. 25%). Respondents in the intervention community were also more likely to report condom use with the most recent new partner in 2010 compared to the control community (Figure 1) and compared to the baseline (p < 0.001). However, there was a considerable increase in reported condom use with the previous new partner in the control community too (p = 0.054). The percentage stating that they did not use a condom because they did not have one at hand dropped in the intervention community (p = 0.041), whereas there was no significant change in the control community. The proportion of respondents who believed that condoms were very or somewhat effective in preventing STIs and HIV increased in both communities (p = 0.001 in the intervention and p < 0.001 in the control area). In the follow-up survey respondents in the intervention community were more likely to report having experienced HIV preventive activities in the venue where they were interviewed and having discussed HIV prevention with a peer educator in the previous 6 months than respondents in the control community (Table 4). However, there was no association between reported condom use and having talked to a peer educator (results not shown).

**Table 4:** Behaviour, perceptions and prevention-related experiences of guests in venues where people meet new sexual partners

		Baseline					Follow-up				
		**Intervention**		**Control**			**Intervention**		**Control**		

		**%**	***N***	**%**	***N***	**p-value**	**%**	***N***	**%**	***N***	**p-value**

Come to meet sexual partner		53	*150*	58	*267*	0.649	29	*224*	32	*211*	0.720

Ever met sexual partner here		70	*180*	66	*326*	0.453	55	*260*	54	*264*	0.792

Condom last time partner from here		57	*116*	55	*217*	0.745	82	*126*	68	*123*	0.075

How often condom w/new partner last month	Always	52	*135*	37	*249*	0.050	66	*118*	52	*147*	0.102
	Sometimes	41		46			21		20		
	Never	7		17			13		29		

Paid for last condom							22	*170*	72	*170*	< 0.001

Condom with you?		39	*178*	32	*325*	0.277	11	*243*	7	*257*	0.289

Condom shown if claimed to have brought		94	*79*	90	*114*	0.350	100	*21*	96	*14*	0.189

How effective are condoms	Very	51	*178*	38	*325*	0.054	67	*264*	57	*272*	0.096^1^
	Somewhat	17		22			17		23		
	Not very	25		26			6		12		
	Not at all	7		7			5		6		
	Don't know	1		8			5		1		

At risk of HIV	No	32	*179*	36	*325*	0.211	41	*264*	36	*271*	0.736
	Moderate	40		33			34		40		
	High	21		18			18		16		
	Very high	6		14			7		8		

Discussed with anyone how to prevent infection		81	*178*	75	*324*	0.391	71	*263*	66	*271*	0.372

If yes, with whom	Parents	4	*148*	0.4	*249*	0.012	1	*197*	2	*184*	0.823
	
	Spouse	41	*148*	39	*249*	0.783	19	*197*	29	*184*	0.105
	
	Friends	85	*148*	81	*249*	0.458	72	*197*	65	*184*	0.328
	
	Peer educators	11	*148*	21	*249*	0.057^1^	55	*197*	6	*184*	< 0.001
	
	Health personnel	24	*148*	31	*249*	0.361	22	*197*	28	*184*	0.299

Ever experienced any HIV preventive activities in this venue							62	*242*	16	*257*	< 0.001

^1^Significant difference when adjusted for type of site and age and gender of respondents

In both communities respondents reported less new partners and less partners overall in the previous month in the follow-up than in the baseline survey (Table 5), and there was a decline in the proportion of men and women engaging in transactional sex in the previous 3 months (85% to 65% of women (p = 0.050) and 87% to 43% of men (p < 0.001) in the intervention community). Women who admitted exchanging sex for money, were more likely to report having used a condom the previous time in the follow-up than in the baseline survey (the increase was only significant in the intervention community: 54% to 80%; p = 0.043).

**Table 5:** Differences in reported number of partners by guests socializing in venues where people meet new sexual partners

	Baseline							Follow-up						
	**Intervention**			**Control**				**Intervention**			**Control**			

	**Est**.	***N***	**SD**^1^	**Est**.	***N***	****SD**^1^**	**p-value**	**Est**.	***N***	****SD**^1^**	**Est**.	***N***	****SD**^1^**	**p-value**

Median number sex partners last 4 weeks	3	*177*		3	*323*			1	*260*		1	*273*		

Mean number sex partners last 4 weeks	3.04	*177*	1.85	3.16	*323*	2.19	0.200	2.30	*260*	19.1	2.04	*273*	5.22	0.443

Median number **new **sex partners last 4 weeks	2	*177*		2	*323*			0	*254*		1	*268*		

Mean number **new **sex partners last 4 weeks	1.75	*177*	1.46	2.09	*323*	1.83	< 0.001	1.20	*254*	4.19	1.78	*268*	5.03	< 0.001

Median number sex partners last 12 months								2	*256*		2	*262*		

Mean number sex partners last 12 months	-	-	-	-		-		5.26	*256*	21.3	7.61	*262*	16.9	< 0.001

^1^Median, mean and SD of mean are not adjusted for clustering

## Discussion

The comparison of the two PLACE-surveys conducted in two townships in Livingstone in 2005 and 2010 indicated clear improvements over the five year period in condom availability and outreach of peer education activities in venues where people meet new sexual partners, particularly in the intervention community. In addition, interviews with people socializing in the venues indicated marked changes in their sexual behaviour. There were decreases in the reported number of sexual partners and the proportion reporting engaging in transactional sex and increases in reported condom use in both communities. However, the increase in reported condom use with the previous partner met in the venue was particularly sharp in the intervention community where most of the respondents reported obtaining the condom in the same venue. It thus seems likely that the increase in reported condom use in high risk places in the intervention community was partially due to the condom distribution and peer education intervention in these places.

Based on previous research there is mixed evidence to back up the hypothesis that targeting HIV preventive activities at high risk places has an impact on the behaviour of people socializing there. A randomised controlled trial in Nicaragua, found that providing condoms in motels lead to increased condom utilisation among guests, but the presence of leaflets and posters promoting condoms did not [5]. PLACE-surveys in East London, South Africa, found increased reporting of condom use and a reduction in multiple partnerships among guests socializing in venues where people met new sexual partners over a three year period, and this could possibly be attributed to a behaviour-change intervention targeting these venues [13]. However, a randomised controlled trial conducted in Kingston, Jamaica, where venues where people met new sexual partners were randomised to a site-based intervention (including on-site HIV testing, condom promotion, and peer education), found no significant differences in reporting of number of partners or consistent condom use between guests in intervention and control venues. However, there were several factors that possibly could explain the lack of impact: implementation difficulties (condoms and educational materials not always being available in the intervention venues), spill-over effects due to patrons visiting both intervention and control venues, national HIV prevention campaigns, time-gap between the intervention and the post-intervention survey, and other interventions being run in some of the control venues [14].

The overall objective of this targeted condom distribution and peer education intervention was to reduce the incidence of HIV and other STIs, but we did not measure any biological outcomes of the intervention. Measuring effects on incidence of HIV in a population requires a much bigger sample size and investment. Very few other intervention studies targeting high risk places have attempted to do so. However, reported STI cases indicate a decreasing prevalence of STIs overall in both communities during the intervention period, and the relative decline in reported cases was sharper in the intervention than the control community (38% vs. 16% from 2008-2010) (clinic registries of the public clinics serving the intervention and control communities). The sharper decline in Maramba may be partially due to our intervention, but any attribution must be done with caution. A study in Zimbabwe with a much stronger design, a cluster-randomised trial, which included a peer education and condom distribution component, did not find any impact on HIV and STI incidence [15].

Condom distribution to high risk places may obviously be organized in different ways, and using peer educators to do this is possibly not the cheapest (at least if the peer educators are provided financial compensation) and quickest way. Nonetheless, using peer educators provides an additional opportunity to engage people in discussions about HIV prevention. Studies indicate that peers of the same sex are an important source of information about sex-related issues among young people [16]. It is likely that a higher number of individuals socializing in the venues would have been reached with HIV-related information in this study if the peer educators had been available when the venues were busier. However, it is not possible to distinguish whether the increased reporting of condom use among guests was a result of the combination of condom distribution, condom demonstrations and behaviour change discussions conducted by peer educators or of improved condom availability alone. People who had been in contact with a youth peer educator were not more likely to report using condoms. On the other hand it seems likely that the peer educators would have had a bigger impact if persons of different ages had been recruited although the evidence for peer educator effectiveness from other studies again is mixed. A randomised controlled peer education intervention study among male beer hall patrons in Zimbabwe, which included condom information and demonstrations and recruitment of men of different ages, did not find any impact of the intervention on unprotected sex with non-marital partners [17], and a review of youth peer education intervention studies conducted between 1998 and 2005 found no impact on condom use [18]. Nonetheless, a review of studies on youth peer education interventions for HIV prevention in low- and middle-income countries conducted between 1994 and 2008 found that such programs often resulted in increased HIV-related knowledge and increased reporting of condom use, but that there was less evidence for an effect on sexual abstinence and number of partners [19].

It is likely that lack of knowledge among new staff explained why not all representatives interviewed in the intervention venues in the follow-up survey reported that condom distribution had taken place there. At the same time, the external monitor revealed that continuous availability of condoms was not fully achieved although this was one of the most important objectives of the intervention. It was expected that the peer educators would need some time to sort out the demand for condoms in the venues, and this may explain why as many as a quarter of the venues lacked condoms in the first monitoring round. Receiving feedback from the monitor probably motivated the peer educators to ensure that they distributed sufficient condoms after this. However, some peer educators seemed to continue to underestimate the demand for condoms, possibly because they did not visit the venue frequently enough, for example in relation to busy weekends.

The improved condom availability in the control community could indicate a trend towards improved condom distribution, i.e. that it was easier for venue owners to obtain free or subsidised condoms that could be given or sold to customers. It is also possible that the increased availability was partly a spill-over effect from the intervention as some of the peer educators lived in the control community and reported that they had distributed condoms in bars and night clubs in their own neighbourhood too since they had not been aware that it would serve as a comparison during the evaluation. Since we found that there was an increase in the proportion who believed that condoms were effective as HIV prevention, it is also possible that venue staff and owners may have experienced an increased demand for condoms from guests, and this may have motivated them to make efforts to offer condoms. The reduction in high risk behaviours reported both among respondents in the intervention and control communities may indicate a general trend. In addition to influence from national campaigns, local prevention efforts carried out by different NGOs in partnership with the DHMT may have had an impact. These have included drama, training of youths, improved VCT and PMTCT services, free provision of condoms and STI treatment services for female sex workers, and promotion of subsidized condoms in high risk places (personal communication with former District Director of Health, Dr. Chinyonga). A behaviour change in the general population would explain the decline in HIV and syphilis prevalence observed among young pregnant women in Livingstone during the period 2002-2008 [4,20] and also be in line with behaviour changes reported in other studies in the region [21-24].

The assignment of the intervention was not randomised. Thus we cannot rule out that there were other important differences between venues and respondents in the intervention and the control community, which were not related to the intervention, but which could explain some of the observed changes. Since there was a rather long period of five years between the baseline and follow-up surveys, it is possible that other programs and changes had taken place in both the intervention and the control community. Fortunately, the adjusted logistic regression analyses indicated that differences in types of venues included in the surveys did not influence the main findings. The low number of refusals in the baseline and the lack of refusals in the follow-up surveys may be an underestimation. Since only two interviewers conducted interviews in the same venue, it would be easy for people to shun them if they were reluctant to be interviewed. Thus the respondents interviewed may not have been representative of all the guests in the venues. If people who were willing to be interviewed were more likely to be consistent condom users, we may have overestimated condom use. In addition, we cannot rule out that the intervention itself made some people change what venues they preferred socializing in. People who knew they were taking high risks might have wanted to avoid places where peer educators talked about HIV and prevention.

## Conclusion

Despite the weaknesses in the design of this condom distribution and peer education intervention, we believe that the presented findings indicate that (almost) continuous condom availability in places where people meet new sexual partners resulted in increased condom use among people who socialized in these venues. The scientific literature is consistent regarding the substantial effect of condom availability on use, suggesting the findings to be highly transferable to a variety of contexts. Thus governments and stakeholders should make specific and dedicated efforts to ensure stable condom availability in places where people meet new sexual partners. The Zambian "National HIV/AIDS/STI/TB Policy" does mention bars as important outlets for condom sale [24], but the implementation needs to be substantially strengthened as shown in the present study. Studies with a randomised cluster design are recommended to obtain a better evidence base relating to the impact of improved condom availability and peer education in high risk places on condom utilization among guests and HIV incidence in the general population.

## Competing interests

The authors declare that they have no competing interests.

## Authors' contributions

IFS conducted the baseline survey, conceived the idea for the intervention study, designed the study, wrote the protocol, designed the evaluation tools, analysed the data, interpreted the findings and drafted the manuscript. CZ coordinated the intervention with the local supervisors, interpreted the findings, and revised the manuscript. CM assisted in designing and planning the intervention, coordinated the evaluation, interpreted the findings, and revised the manuscript. KF interpreted the findings and revised the manuscript. All authors approved the final version of the manuscript.

## Pre-publication history

The pre-publication history for this paper can be accessed here:

http://www.biomedcentral.com/1471-2458/12/10/prepub
